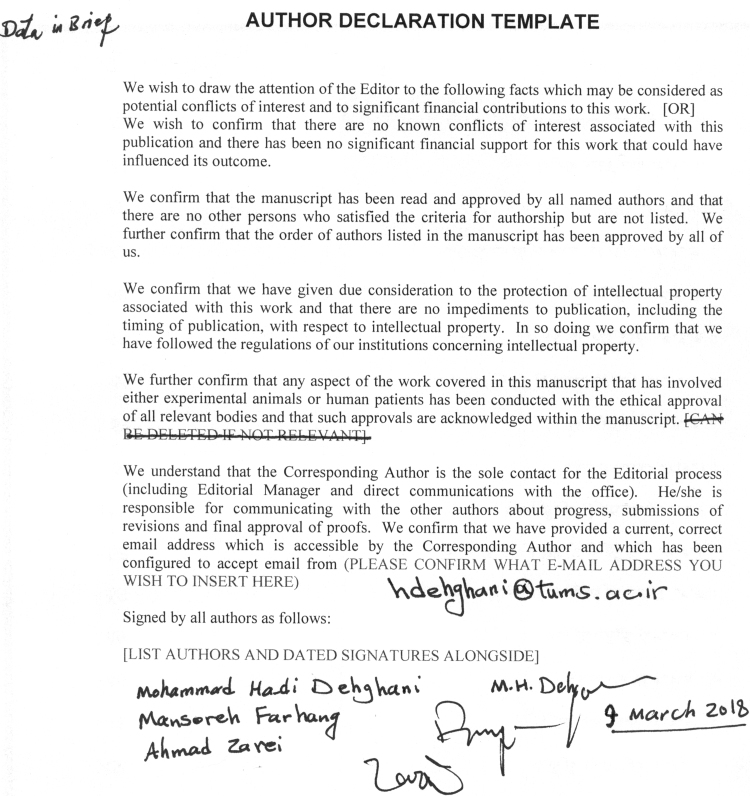# Data on the level of haloacetic acids in indoor swimming pools of Iran: A case study of Tehran

**DOI:** 10.1016/j.dib.2018.05.004

**Published:** 2018-05-09

**Authors:** Mohammad Hadi Dehghani, Mansoureh Farhang, Ahmad Zarei

**Affiliations:** a Department of Environmental Health Engineering, School of Public Health, Tehran University of Medical Sciences, Tehran, Iran; b Institute for Environmental Research, Center for Solid Waste Research, Tehran University of Medical Sciences, Tehran, Iran; c Department of Environmental Health Engineering, School of Public Health, Gonabad University of Medical Sciences, Gonabad, Iran

**Keywords:** Haloacetic acids, Indoor swimming pools, Disinfection, Tehran

## Abstract

Haloacetic acids (HAAs) are the second most prevalent class of DBPs after trihalomethanes (THMs) in water disinfected by chlorine compounds. Within this study, we present new data on occurrence and speciation of HAA levels in 15 indoor swimming pools in Tehran in 2017. Five HAAs (HAA5), including monochloroacetic acid, dichloroacetic acid, trichloroacetic acid, monobromoacetic acid, and dibromoacetic acid were analyzed. Levels of pH, total chlorine, and total organic carbon concentration were analyzed as well. Results indicated that the levels of HAA5 in swimming pools in the Tehran ranged from 148 to 3488 µg/L, with an average of 1045.26 µg/L. HAAs in the swimming pools in Tehran might be due to the extensive use of chlorine compounds for disinfection. Therefore, due to the high levels of HAAs, frequent monitoring of HAA levels as well as minimization strategies is needed in these swimming pools.

**Specifications Table**

**Table:** 

Subject area	Environmental Health Engineering
More specific subject area	Haloacetic acids in swimming pools
Type of data	Table, text file
How data was acquired	Survey, US. EPA Method 552.3, capillary column gas chromatographic method by an electron capture detector
Data format	Raw, analyzed
Experimental factors	The mentioned parameters above, in abstract section, were analyzed according to the Standard Methods for the Examination of Water and Wastewater
Experimental features	Levels of haloacetic acids (HAA) in 15 indoor swimming pools in Tehran were determined.
Data source location	Tehran, Iran, Coordinates: 35.6892°N, 51.3890°E
Data accessibility	The data are available only in this article

**Value of the data**

• Understanding the formation and control of haloacetic acids in indoor swimming pools have important public health consequences considering the widespread use of swimming pools in any nation.
• The formation of the haloacetic acids as common DBPs has triggered a major concern on the human health since their presence in water has been linked to cancer, adverse reproductive outcomes following exposure during pregnancy and hepatic toxicity.
• In this work, five most common Haloacetic acids including monochloroacetic acid (MCAA), dichloroacetic acid (DCAA), trichloroacetic acid (TCAA), monobromoacetic acid (MBAA) and dibromoacetic acid (DBAA), were analyzed in 15 indoor public swimming pools in Tehran.

## 1 Data

Supply of safe water is necessary for human [1], [2], [3], [4]. Water disinfection is necessary for the protection of human health, which significantly diminishes mortality rates and infectious diseases [3], [4], [5], [6]. However, the reaction of disinfectants, especially chlorinated compounds, with natural organic matter, bromide and iodide present in water forms disinfection byproduct (DBPs) which are carcinogen [7]. Haloacetic acids (HAAs) are the one of the most predominant classes of chlorination byproducts and thus are good indicators of the total DBPs in chlorinated water [8], [9]. Due to their potentially deleterious impacts on human health, a great attention is focused on HAAs in recent years, and many nations or international agencies have set regulations to reduce these hazardous materials in water [10], [11], [12], [13].

In this study the levels HAAs and speciation of HAAs were investigated under various operation conditions typically used in Iranian indoor public swimming pools. Analytical results of sum of five most common Haloacetic acids including monochloroacetic acid (MCAA), dichloroacetic acid (DCAA), trichloroacetic acid (TCAA), monobromoacetic acid (MBAA) and dibromoacetic acid (DBAA) in 15 indoor swimming pools in Tehran. The averages for measured TOC and total chlorine in the swimming pools are also are summarized in Table 1.

**Table 1:** Parameters measured in indoor swimming pool water samples.

**Pool name**	**pH**	**Water temperature (C**^**o**^**)**	**Turbidity (NTU)**	**TOC (mg/l)**	**Total chlorine(mg/l)**	**Number of users per hour (average)**
A	7.5	25	0.23	5	2	12
B	7	23	0.34	1.7	1.5	14
C	7.8	27	0.6	2.4	3	17
D	8.2	24	0.46	1.1	5	8
E	7.9	24	0.56	15	1	35
F	7.3	27	0.36	3	1.5	11
G	7.1	23	0.35	9.8	3.5	22
H	7.7	26	0.54	15.5	0.8	32
I	7.5	27	0.31	4.4	4	10
J	7.2	29	0.44	12.6	0.8	28
K	8.1	28	0.41	6.3	2.5	12
L	7	26	0.37	3.9	3	9
M	7.9	27	0.45	7.1	1	13
N	8.4	25	0.32	17	0.7	41
O	7.3	24	0.31	1.9	2.5	9

## 2 Sampling protocol, HAAs extraction and HAA analysis

15 pools most crowded indoor swimming pools were selected, sampled and analyzed for the occurrence of HAAs. Totally 30 samples (2 samples from each pool) were collected at the water depth of 15 cm. This study was conducted during September 2017. The samples were taken immediately after swimming. Prior to sampling, ammonium chloride was added to the sample bottles to convert the free chlorine residual in the water to combined chlorine. Total chlorine, pH and total organic carbon (TOC) concentration were analyzed as well. All the samples were collected using 250 ml amber glass bottles. These bottles were filled and then tightly sealed with Teflon lined screw caps. The bottles were taken to the laboratory within 4 h, then refrigerated at 4 °C and kept in absence of light until extraction. Sample analysis was done within one week of collection date. In this study, the levels of HAAs in influent water of pools were not determined. The concentrations of HAAs were determined in accordance with US. EPA Method 552.3 with some slight modifications[14]. At the beginning, a volume of 40 mL of sample was acidified by using 2 mL of 98% Sulfuric acid to decrease pH below 0.5. To achieve phase separation and a saturated solution, 2 g CuSO_4_ and 16 g Na_2_SO_4_ were used, separately. The HAAs extraction was done by using 4 mL MTBE followed by 30 min agitation. Then, 3 mL of MTBE with 1 mL acidic methanol was heated at 50 °C for 1.5 h to derive HAA and the extract was cooled down immediately. In order to naturalize the solution, saturated NaHCO_3_ was used and agitated completely before CO_2_ ventilation. Then, it was phase separated for 1 min and 1 mL of methylated HAAs was extracted into a 2 mL vial. The methylated HAAs were measured using gas a chromatography-mass spectrometry [15]. Five HAAs (HAA5), including dichloroacetic acid, monochloroacetic acid, trichloroacetic acid, monobromoacetic acid, and dibromoacetic acid were measured. For quality assurance/quality control (QA/QC), QC check standards and matrix spikes, were used during the analysis. The detection limits for the analyses of the studied HAA5 species including MCAA, DCAA, TCAA, MBAA, and DBAA were 0.273, 0.242, 0.079, 0.204, and 0.066 μg/L, respectively. For determination of total organic carbon (TOC), the collected samples were conveyed to forty mL glass vials, acidified with H_3_PO_4_ to pH=2, and stored at 4 °C for analysis. The values of total chlorine and pH were determined at the sampling sites.Total organic carbon (TOC) were measured using a Shimadzu TOC analyzer (model ASI 5000) [16]. All statistical analyses were performed with SPSS software version 22.

## 3 The diagram of conventional pool water treatment systems

**Fig. 1:**
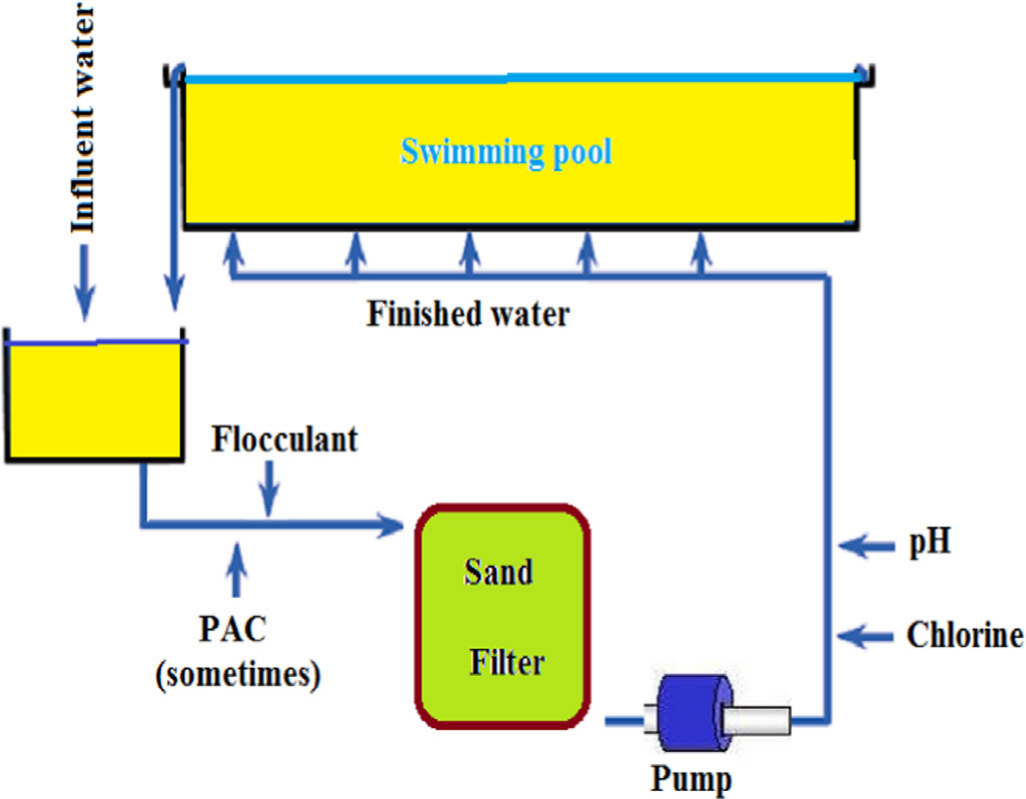
The diagram of a conventional pool water treatment system.

The processes used for water treatment in swimming pools in Tehran are given in Fig. 1.

## 4 Parameters measured in indoor swimming pool water samples

The key parameters affecting the levels of HAA5 are given in Table 1.

## 5 Levels of HAAs in indoor swimming pools

Levels of Trichloroacetic acid (TCAA), dichloroacetic acid (DCAA), monochloroacetic acid (MCAA), monobromoacetic acid (MBAA) and dibromoacetic acid (DBAA) as well as HAA5 are given in Table 2.

**Table 2:** HAAs concentrations (μg/l) in indoor swimming pool water samples.

	**TCAA**	**DCAA**	**MCAA**	**MBAA**	**DBAA**	**HAA5**
A	368.28	211.42	73.65	17.75	10.88	682
B	536.59	510.45	134.46	41.73	21.76	1245
C	108.1	91.65	25.38	5.94	3.92	235
D	72.52	47.36	15.98	8.76	3.37	148
E	1264.64	753.92	262.65	113.81	36.96	2432
F	539	363	118.8	58.3	20.9	1100
G	1709.12	1255.68	376.70	90.82	55.66	3488
H	176.8	98.6	36.72	21.42	6.46	340
I	382.7	364.9	96.12	30.72	15.55	890
J	479.71	342.65	105.73	30.07	20.83	979
K	190.08	133.92	46.65	51.49	9.84	432
L	589.6	549.4	144.72	34.89	21.38	1340
M	563.75	577.5	148.5	58.08	27.17	1375
N	164.73	106.59	34.88	10.65	6.137	323
O	361.8	201	72.36	24.65	10.18	670

Trichloroacetic acid (TCAA), dichloroacetic acid (DCAA), monochloroacetic acid (MCAA), monobromoacetic acid (MBAA) and dibromoacetic acid (DBAA).

## Figures and Tables

**Fig. S1:**